# A Combination of FPU-Net and Feature Clustering Methods for Accurate Segmentation of Femoral Neck in Radiographic Diagnosis

**DOI:** 10.3390/diagnostics13172855

**Published:** 2023-09-04

**Authors:** Y. Y. Chen, C. H. Chuang, S. L. Hsieh, T. L. Lin, C. J. Hsu

**Affiliations:** 1 Department of Artificial Intelligence and Computer Engineering, National Chin-Yi University of Technology, Taichung 411030, Taiwan; vpcyy2233@gmail.com (Y.Y.C.); chchuang@ncut.edu.tw (C.H.C.); 2 Department of Orthopedic Surgery, China Medical University Hospital, Taichung 404327, Taiwan; alilin422@yahoo.com.tw (T.L.L.); d5983@mail.cmuh.org.tw (C.J.H.)

**Keywords:** segmentation, femoral neck, computer-aided diagnosis, radiograph, deep learning

## Abstract

In this study, we develop an innovative method that assists computer-aided diagnosis in the determination process of the exact location of the femoral neck junction in plain radiographs. Our algorithm consists of two phases, i.e., coarse prediction and fine matching, which are implemented by supervised deep learning method and unsupervised clustering, respectively. In coarse prediction, standard masks are first produced by a specialist and trained in our proposed feature propagation network (FPU-Net) with supervised learning on the femoral neck dataset. In fine matching, the standard masks are first classified into different categories using our proposed three parameters with unsupervised learning. The predicted mask from FPU-Net is matched with each category of standard masks by calculating the values of intersection of union (IOU), and finally the predicted mask is substituted by the standard mask with the largest IOU value. A total of 4320 femoral neck parts in anterior–posterior (AP) pelvis radiographs collected from China Medical University Hospital database were used to test our method. Simulation results show that, on the one hand, compared with other segmentation methods, the method proposed in this paper has a larger IOU value and better suppression of noise outside the region of interest; on the other hand, the introduction of unsupervised learning for fine matching can help in the accurate localization segmentation of femoral neck images. Accurate femoral neck segmentation can assist surgeons to diagnose and reduce the misdiagnosis rate and burden.

## 1. Introduction

It has been reported that hip fractures account for about 20% of all orthopedic hospitalizations with a global annual incidence of 4 million hip fractures in 2025 and 6.3 million in 2050 [1,2]. The frequency of this disease will increase significantly in affluent parts of the world where aging societies are accelerating. Femoral neck fractures account for approximately half of all hip fractures and are one of the most common injuries in the elderly population and result in high-financial burden [3,4]. Femoral neck fractures in patients under the age of 60 are usually due to high-energy trauma, which poses challenges for surgical treatment and only a small number of patients can return to their pre-injury activity level [5,6]. Somehow, imaging misdiagnosis of non-displaced femoral neck fractures may occur in non-orthopedic surgeons or young radiologists in the emergency department. In order to avoid this situation, we propose a new method to help the medical staff in the first line to automatically identify the femoral neck location in radiographs. Moreover, we aim to identify the nondisplaced femoral neck fracture lines with this method in the next steps.

A radiographic anterior–posterior (AP) pelvis view is the common diagnostic approach for fractures diagnosis. However, the sensitivity of radiographic examination of femoral neck fractures is not ideal since there are a number of technical factors that can affect our ability to accurately obtain medical information, such as penetration, aspiration, rotation, magnification, angulation, inclusion, and artifacts. In fact, many hospitals lack experienced surgeons and radiologists to diagnose fractures with statistics reporting a misdiagnosis rate of between 7% and 14% for initial emergency room visits [7]. As a result, computer-aided diagnosis is being developed to assist surgeons in their diagnosis in order to reduce the burden and misdiagnosis rate.

In recent years, deep convolutional neural networks (DCNN) have been rapidly developed and are widely used in the medical field including image segmentation, classification and so on, which help to obtain accurate diagnosis results, reduce medical errors, and improve work efficiency [8,9,10,11,12]. DCNNs learn image features from data automatically and are self-tuned by back propagation using multiple building blocks such as convolutional, pooling and fully connected layers [13]. Our study focuses on the segmentation of the ROI (region of interest) of the femoral neck. Any degree of image distortion in the radiographs will distort the ROI region of the femoral neck in the hip images and lead to misinterpretation. Intensity variations in grayscale values, abnormal image rotation, and disproportionate magnification of the femoral neck junction are the most common image distortions when hip radiographs are taken [14,15,16]. Previous automatic DCNN-based segmentation methods, such as fully convolutional networks [17], PSPNet [11], U-net and its improved methods [18,19,20], have been proposed to segment medical images. However, the detection of femoral neck ROI regions is a challenging task, especially since radiograph images often carry the image distortions as mentioned above.

Therefore, we propose a new method combining supervised deep learning and unsupervised learning strategies to precisely segment the region of femoral neck junction and mark the ROIs prior, in order to further assist surgeons in computer-aided diagnosis. The method is implemented in two steps: coarse prediction and fine matching. In the coarse prediction phase, a corresponding mask is created in collaboration with specialized orthopedic surgeon, and a limited data set of the original radiographs is augmented by rotation and scaling to simulate various image distortions. A new feature propagation pathway was added to the classical U-Net as a propagation pathway for collecting image features in the femoral neck ROI region and denoted as FPU-Net. In the fine matching step, the masks made by the orthopedic surgeon are used as criteria for classification using unsupervised learning methods and finally matched with the masks of the tests. The matching criterion is to calculate the intersection of the union (IOU) [21] value between each tested mask and standard mask category. The standard mask category corresponding to the largest IOU value is selected to replace the test mask as the basis for segmenting the femoral neck junction. We developed an innovative method that assists computer-aided diagnosis in the determination process of the exact location of the femoral neck junction in plain radiographs. Accurate femoral neck segmentation can assist surgeons to diagnose and reduce the misdiagnosis rate and burden.

## 2. Materials and Methods

In this section, the network structure of our proposed FPU-Net is introduced, followed by the combination of FPU-Net and unsupervised clustering methods. The radiographic images were acquired from 240 patients between the years 2018 and 2020. Two senior orthopedic surgeons were involved to label the femoral neck part. Images for all experiments were de-identified and collected from China Medical University Hospital, Taichung, Taiwan. There was no interaction with patients directly, as we acquired de-identified data. This study was in accordance with the ethical standards of the institutional and national committee on human experimentation, and was conducted according to the guidelines of the Declaration of Helsinki. All the labeled images used in FPU-Net were produced by a professional orthopedist and radiologist. To reduce computation time, the left and right femoral neck were rescaled to new images of 1024 × 1024 pixels, which were then stored as the initial dataset. The initial dataset was data augmented by rotation and scaling strategy, resulting in a total of 4320 images. Half of these images, i.e., 2160 images, were used as training data, and the rest were used for the prediction of the trained DCNN.

**Figure 1:**
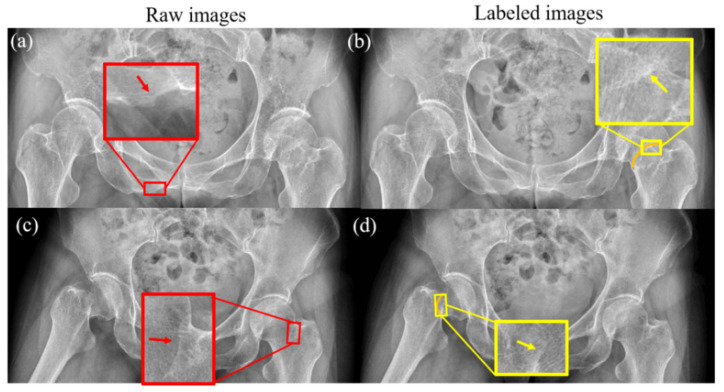
Healthy bone tissue and femoral neck fractures have similar grayscale distributions. The grayscale distribution of normal bone is shown by the red arrows in (**a**,**c**), and the grayscale distribution of the fracture region is shown by the yellow arrows in (**b**,**d**). The orange lines in (**b**,**d**) are the fractures of femoral neck labeled by a surgeon.

The DCNN was trained to obtain features in the region of femoral neck, based on the gray distributions. However, the boundary regions of healthy bone tissue in radiographs may show a gray distribution similar to that of a fracture, as illustrated in Figure 1. Raw image and its labeled image with orange curves made under the guidance of a professional orthopedist and radiologist are shown in the left and right column, respectively. As shown in the enlarged views in the red boxes of Figure 1a,c and that in the yellow boxes of Figure 1b,d, the similar gray regions indicated by the arrows may affect the segmentation accuracy of the femoral neck fracture. Hence, training the full frame image for DCNN to segment the fracture regions may mislead the process, which may also degrade the performance of the network. In order to improve the performance of DCNN, an effective method is needed to exclude these interferences.

### 2.1. Network Architecture of FPU-Net

**Figure 2:**
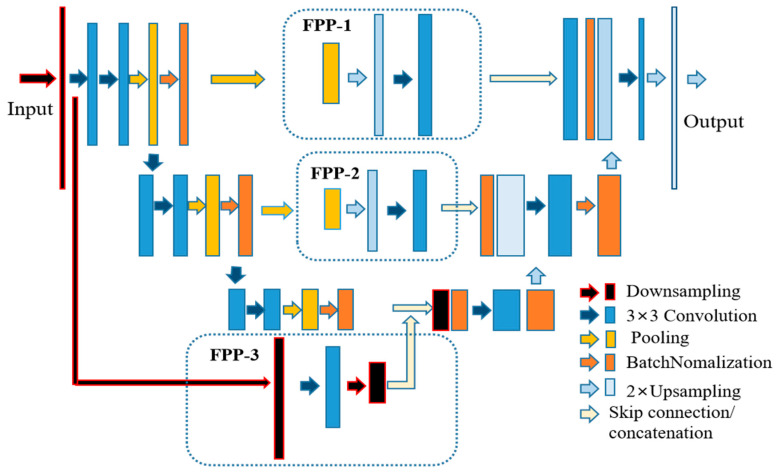
Schematic diagram of our proposed FPU-Net. The feature propagation pathways (FPP) shown in the dotted box is added to the U-Net to obtain more image features. FPP-1 consists of a sequence of 8 × 8 pooling, 8 × 8 upsampling and 3 × 3 convolutional layers. FPP-2 consists of a sequence of 16 × 16 pooling, 16 × 16 upsampling and 3 × 3 convolutional layers. FPP-3 consists of a down-sampling layer, a 3 × 3 convolutional layer and a 2 × 2 down-sampling layer.

The network architecture is illustrated in Figure 2, which consists of a classical U-Net and a newly designed feature propagation pathway. The encoder part of the FPU-Net on the left employs a downsampling layer to resize the original input image, followed by the main convolution block, which consists of a sequence of two 3 × 3 convolutional layers, an average pooling layer with a stride of 2 and a pooling size of 2 × 2, and a batch normalization layer. The main block is repeated three times and the number of the convolution kernels is doubled after each downsampling step to produce more output features. The detailed parameters of the encoder are shown in Table 1.

Three pathways, named feature propagation pathway (FPP), were added to the network architecture to supplement more hip radiograph features as shown in the dashed boxes in Figure 2. FPP-1 consists of a sequence of 8 × 8 pooling, 8 × 8 upsampling, and FPP-2 with a double size of pooling and upsampling, and a 3 × 3 convolutional layer is added to both FPPs to effectively reduce the aliasing effect of upsampling. FPP-3 consists of a downsampling layer, a 3 × 3 convolutional layer, and a 2 × 2 downsampling layer. The utilization of the FPPs allows the network to extract multiscale features and deliver them to the decoder. The detailed network parameters of FPP are shown in Table 2.

The decoder in the right part of the network architecture is used to reconstruct high-resolution data from lower resolution data. The detailed parameters of the decoder are shown in Table 3. The decoder part is followed by a block that consists of a 3 × 3 convolution operation and a batch normalization layer, repeated three times. The inputs to the decoder bottom layers are the concatenation of the encoder and the feature propagation pathway. A upsampling layer is used after each block to obtain high-resolution information and to reduce the channels of the feature maps. Finally, a 1 × 1 convolution filter and an upsampling operation is performed to produce the final results. Compared with other classical DCNNs, the feature propagation pathways in FPU-Net can obtain more image features of hip radiographs, as will be demonstrated in the experimental and results section.

### 2.2. Combine FPU-Net with Unsupervised Method

**Figure 3:**
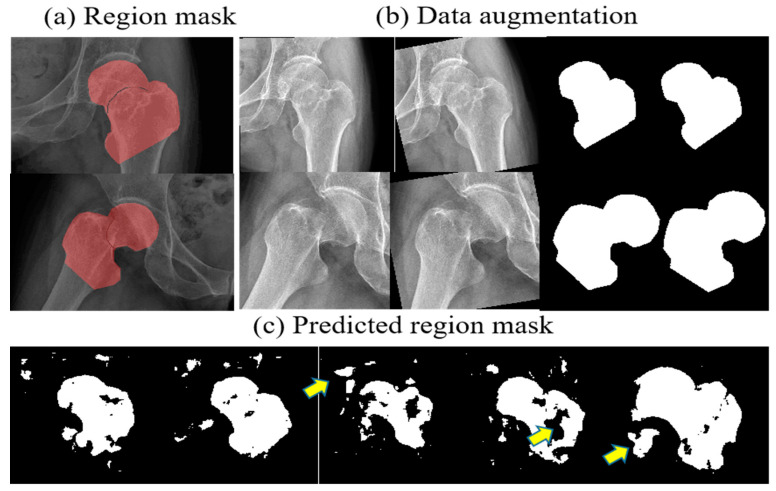
Data augmentation and coarse prediction by FPU-Net. (**a**) binary masks of the femoral neck junction, (**b**) data augmentation, (**c**) predicted mask with FPU-Net. Additional or missing regions can be seen as indicated by the yellow arrows, which affect the accuracy of FPU-Net prediction due to the presence of grayscale noise.

Another challenge that was raised in the radiograph was that the raw radiographic is usually about 4000 × 3000 pixels in size, which will additionally consume a lot of computing resource; at the same time, the region of the femoral neck segmented from the full frame image is also affected by grayscale noise in other regions, which affects the cutting accuracy. In the training of FPU-Net, the mask of the binary region was first produced and used as labels. Due to the shortage of medical data, both the radiograph and its mask were rotated or scaled to increase the training data, as shown in Figure 3.

However, as presented in Figure 3c, the resulting region masks predicted by the well-trained FPU-Net are not as accurate as the standard mask produced by the orthopedic surgeon. Additional or missing regions are shown as indicated by the yellow arrows, which affect the accuracy of FPU-Net prediction due to the presence of grayscale noise. Therefore, FPU-Net combines post-processing methods to solve the problem that grayscale noise affects prediction. The size, orientation or position of bones in the X-ray image may vary depending on technical factors, as well as the physical shape of the imaged object or the patient’s lying position. Under the guidance of a professional orthopedic surgeon, a mask for the femoral neck region can be made in advance according to different imaging cases, i.e., male or female, lean or obese, and different lying positions, and then the predicted results of FPU-Net can be adjusted by the standard masks made. These standard masks are then classified into certain categories based on unsupervised clustering methods.

The clustering method is considered as the most important concept in the unsupervised learning principle, which locates a structure for the collection of unlabeled data [22], and collects objects with “similarity” or “dissimilarity” to become target clusters [23]. 

In this paper, the clustering vector consists of three parameters, namely, area, gray gravity and angle, calculated from the secondary moment of the regions, which represent the size, the position and the orientation of a mask, respectively. The gray gravity $\left(u^{\prime },v^{\prime }\right)$ is defined by the following formulation [24].

(1) $$\left\{\begin{array}{c} u^{\prime }=\sum_{\left(u,v\right)\in \Omega }u\cdot f\left(u,v\right)/\sum_{\left(u,v\right)\in \Omega }f\left(u,v\right)\\ v^{\prime }=\sum_{\left(u,v\right)\in \Omega }v\cdot f\left(u,v\right)/\sum_{\left(u,v\right)\in \Omega }f\left(u,v\right) \end{array}\right.$$

where $f\left(u,v\right)$ is the gray value of a pixel at coordinate $\left(u,v\right)$ of an image and Ω is the set of target regions. To calculate the orientation of a mask, three secondary moments are defined from formulations $M_{20}=\sum_{u}\sum_{v}u^{2}\cdot f\left(u,v\right)$, $M_{02}=\sum_{u}\sum_{v}v^{2}\cdot f\left(u,v\right)$, $M_{11}=\sum_{u}\sum_{v}u\cdot v\cdot f\left(u,v\right)$. Hence, the angle can be calculated from the following formulation:

(2) $$\theta =\frac{1}{2}arctan(\frac{2b}{a-c}),\theta \in [{-90}^{{}^{\circ}},90^{{}^{\circ}}]\;\mathrm{where}\;a=\frac{M_{20}}{\sum_{u}\sum_{v}f\left(u,v\right)}-{u^{\prime }}^{2},\;b=\frac{M_{11}}{\sum_{u}\sum_{v}f\left(u,v\right)}-u^{\prime }v^{\prime }\;\mathrm{and}\;c=\frac{M_{02}}{\sum_{u}\sum_{v}f\left(u,v\right)}-{v^{\prime }}^{2}$$

**Figure 4:**
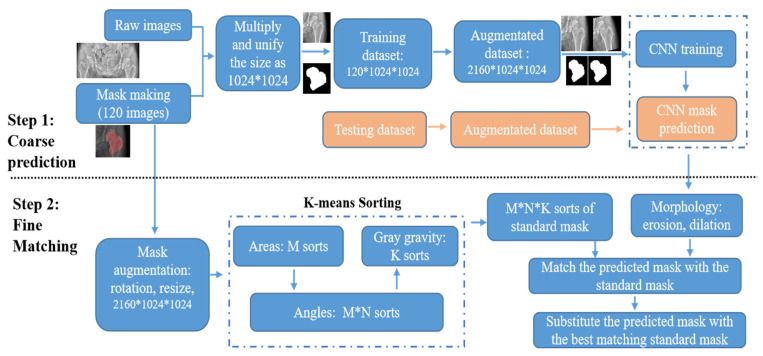
Flow chart of coarse prediction and fine matching pathway. In coarse prediction, the trained FPU-Net model is used to predict the mask of the testing images. In fine matching, standard masks are categorized into certain categories and then the predicted masks are replaced with the best matching standard mask.

The proposed algorithm, including coarse prediction and fine matching steps, is illustrated in Figure 4. The above pathway uses FPU-Net for coarse prediction, and the second pathway below first classifies the standard masks into certain categories and then matches them with the coarse-predicted mask. The standard mask with the largest *IOU* value will replace the coarse-predicted mask in the above pathway. The *IOU* value was calculated according to expression (3) to evaluate the algorithm.

(3) $$IOU=\frac{A\cap B}{A\cup B}$$

where *A* and *B* denotes the areas of the coarse-predicted mask and the standard mask.

In the coarse prediction, the binary standard mask is created to cut out the target region from the raw image. The target image is preprocessed to a unified size of 1024*1024 and then fed into the FPU-Net for training. The trained FPU-Net model is then used to predict the mask of the testing images. Each predicted mask is matched with the sorted subtypes and the correct index of the accurate mask is obtained. In the fine matching, no additional labels are placed on the patient’s radiograph and the standard mask is automatically processed by the K-means algorithm, including the three parameters mentioned above. The predicted coarse masks are preprocessed with morphology erosion and dilation to remove spurious signals, and then the IOU values of the preprocessed masks with each standard mask are calculated, i.e., a total of M*N*K IOU values. The standard mask of the index, corresponding to the largest IOU value, is picked, and this best-fit standard mask replaces the coarse-predicted mask to increase the cutting accuracy. First, the standard masks are divided into M categories according to area, and then each category is further divided into N categories according to angles. Finally, each M*N category is further divided into K categories based on gray gravity, and there are M*N*K categories of standard masks in total.

In summary, we implemented the Algorithm 1 as follows:
**Algorithm 1: Steps of Coarse Prediction and Fine Matching.****Step One: Coarse Prediction** (1)Make binary standard masks and cut the targeted regions out of the raw images;(2)Augment the dataset and train FPU-Net;(3)Predict the mask of the testing images;**Step Two: Fine Matching** (1)Categorize the standard masks into certain subtypes with k-means:(a)Categorize the standard masks into M Categories according to their areas,(b)Further categorize the M Categories into M*N according to their angles,(c)Finally, obtain M*N*K Categories with gray gravity of (b);(2)Match the predicted masks in Step One with the sorted M*N*K subtypes of standard mask;(3)Substitute the predicted mask with the best matching standard mask.

Since the K-means method is the unsupervised learning approach, the input samples can simply be classified into any sorts by setting the parameters of M, N and K. Meanwhile, the more sort numbers, the more accurate the matching result will be, but it also requires more computation time. Therefore, we should balance between the sort numbers and the running time accordingly.

## 3. Results

Previous state-of-the-art networks, such as U-net [18] and PSPNet [11], are used as comparison methods, and for fair comparison, we used the original codes released by the authors. We expanded our dataset by rotating (±10 degrees) and scaling (5%) the radiographs of the femoral neck obtained from China Medical University Hospital to obtain a total of 4320 anterior-posterior (AP) radiographs of the femoral neck to test our method.

### 3.1. Results of Coarse Prediction

**Figure 5:**
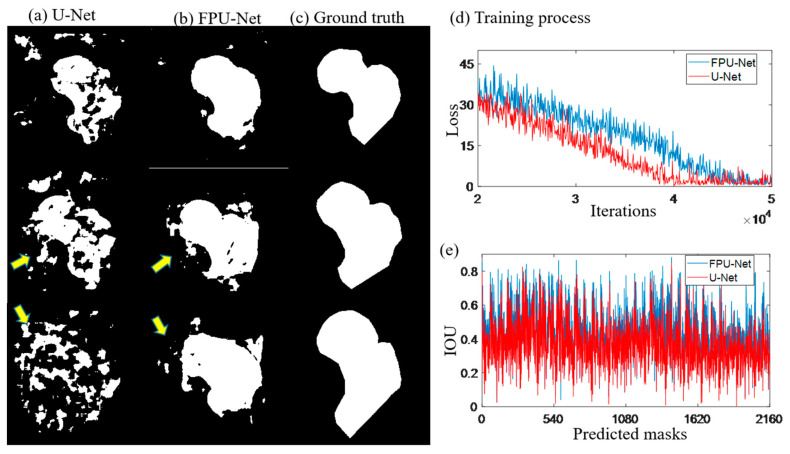
Predicted masks of U-Net and FPU-Net methods. (**a**) U-Net, extra regions were generated as shown by the yellow arrows, (**b**) FPU-Net, more smooth results were obtained and less extra regions were visible, (**c**) ground truth, standard masks made under the guidance of professional orthopedist, (**d**) training process, two methods have similar convergence tendency, (**e**) IOU, calculated the predicted results with the ground truth (the standard mask), respectively, which demonstrated that the FPU-Net method was more accurate.

The network architectures of U-net and FPU-Net share the same layers and channels, except for FPU-net, which has three feature propagation pathways to focus on the details of bone boundaries. The learning rate of both methods was set to 10^−4^ and the loss function was mean absolute error and the optimizer was Adam. In Figure 5d, the U-Net shows a relatively faster convergence, but both networks reach similar convergence after 50,000 iterations for a training batch size of 16.

Figure 5a,b show the predicted results of the U-net and the coarse prediction results of the proposed FPU-Net method, respectively. Figure 5c shows the ground truth (the standard mask) made under the guidance of professional orthopedist. Compared with ground truth, U-net predicts significantly extra and unconnected regions, while the proposed FPU-Net prediction shows the result of complete femoral neck ROI region, except some tiny regions. The calculation of the IOU value for all 2160 predicted masks in Figure 5e shows that the FPU-Net method achieves higher IOU values than the U-Net, which demonstrates that the FPU-Net performs more effectively in the segmentation of the femoral neck.

### 3.2. Fine Matching with Unsupervised Method of Clustering

Although the FPU-Net achieved an effective improvement in the segmentation of the radiograph of the femoral neck, tiny disconnected regions generated by similar gray distribution outside the target region was still observed in the results. FPU-Net combined with unsupervised learning was used to remove the noise caused by the disconnected regions. The K-means method was firstly employed to classify the standard masks into M*N*K categories. The greater the multiplication of the three parameters is, the more accurate the matching results will be; however, more computing time is required as well. Hence, a balance between the category number and the running time were taken into account accordingly, and M, N, K were set to be 16, 8 and 2 in this work, respectively. Prior to fine matching, successive morphological operations, i.e., erosion and dilation preprocessing, were performed on the coarse predictions. Then, the area, angle and gray gravity of each processed masks were calculated and compared with all classified standard masks to find out the best matched mask with the maximum IOU values. Finally, the processed mask was substituted with best matched mask.

**Figure 6:**
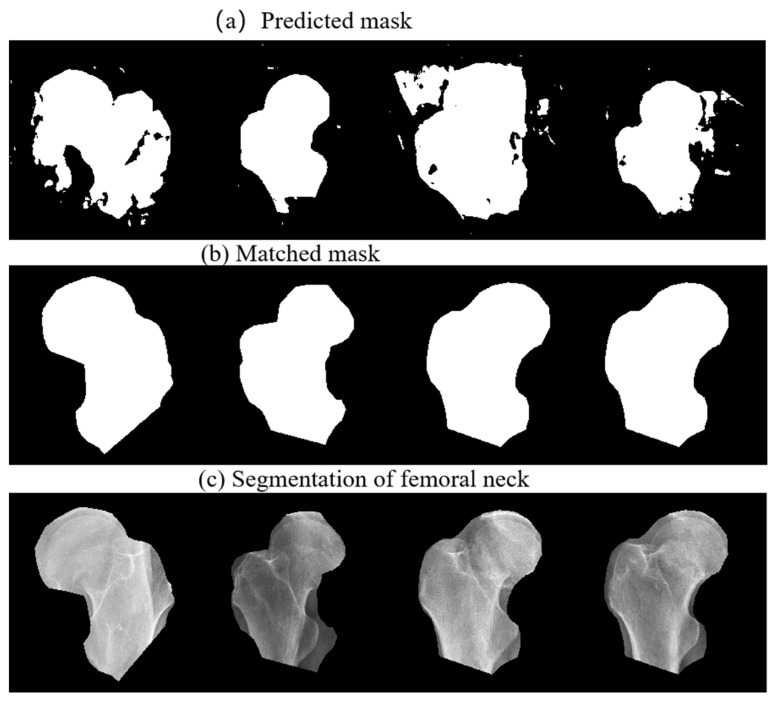
Segmentation of femoral neck using the proposed method. (**a**) Coarse prediction FPU-Net results, (**b**) fine matched FPU-Net results, (**c**) segmentation of the femoral neck using the matched masks.

Figure 6 shows the coarse prediction mask, the fine matching mask, and the final segmentation of femoral neck. In Figure 6a, extra regions, unsmooth boundaries and hollows can be observed in the mask predicted by the coarse prediction FPU-Net. As shown in Figure 6b, after the fine matching FPU-Net, the best matched mask is multiplied with the original radiograph to segment the femoral neck. Figure 6c shows an effective segmentation, where all the boundaries of the masks are closely approaching that of the femoral necks. Hence, we can accurately localize the femoral neck and exclude noise outside the target regions, which helps doctors to discover the fractures quickly in clinical diagnosis, and could be used as a preprocessor for further processing.

**Figure 7:**
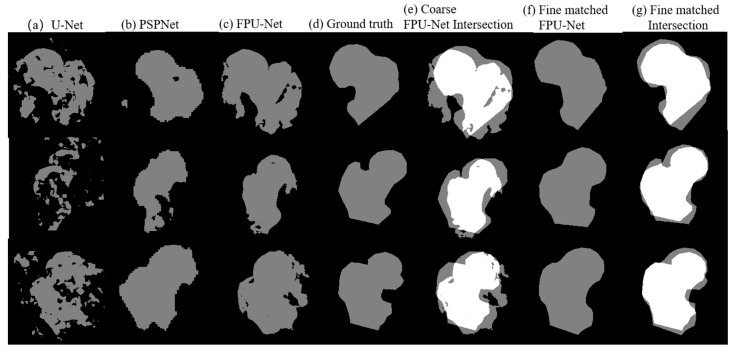
Comparison of the results of the mentioned methods. (**a**) U-Net [18], (**b**) PSPNet [11], (**c**) coarse prediction FPU-Net, (**d**) ground truth produced under the guidance of a professional orthopedic surgeon, (**e**) the intersection area between the coarse prediction FPU-Net and ground truth, (**f**) fine matching of FPU-Net, (**g**) the intersection area between the fine matched FPU-Net and ground truth.

Comparison results of the U-Net, the PSPNet, the coarse prediction FPU-Net and the fine matching FPU-Net are shown in Figure 7. Table 4 calculates the IOU values of the corresponding results for different methods. As mentioned earlier, the binary coarse prediction FPU-Net performs better than the U-Net and PSPNet, as shown in Figure 7a–c. In Figure 7e, the gray region and the white region clearly present the intersection area of the coarse-predicted FPU-Net mask and ground truth. The gray areas indicate the places where the coarse-predicted FPU-Net and ground truth do not overlap. The more these gray areas exist, the worse the prediction results are implied. The mean value of IOUs in Table 4 is below 0.7, which also indicates that the coarse prediction requires further improvement. Comparing the fine matching FPU-Net results with the ground truth, as shown in Figure 7f, the gray regions were obviously reduced, and the boundaries of the fine matched results were approaching the ground truth. The values of IOUs of fine matching FPU-Net in Table 4 is improved to more than 0.8, and the IOUs are improved by more than 25% compared with other methods, which demonstrates the efficiency of the fine matching FPU-Net.

In 2018, M. Z. Alom et al. proposed R2U-Net [25], which combines RNN and Res-Net to transform the structure into a U-net structure; such an architecture has several advantages, allowing residual units to help train the depth framework and allowing the accumulation of features at the base of the cyclic residual volume, ensuring a better representation of segmentation task features. In addition, Oktay, O. et al. also proposed the Attention Gate model [26], which automatically learns the target structures with different shape sizes of interest and integrates them into the U-Net to obtain the Attention U-Net, which can be easily integrated into standard CNN architectures. Q. Zuo et al. later proposed R2au-net [27] to further improve model accuracy. However, all three approaches still require a large number of network parameters and computation time.

We also compared our proposed method with other methods in R2U-Net, Attention U-Net, and R2AU-Net in the literature. The Dice coefficient and Hausdorff Distance metrics were used to evaluate the performance of our proposed method and the other three methods, and the related data are listed in Table 5. As shown in Table 5, although our method is slightly lower than the other methods in terms of IOU, Dice coefficient and Hausdorff distance, the number of parameters of our method is 7,521,953, which is much lower than the other methods and requires 31%, 75% and 92% less resources than the other methods.

The above results shows that our method performs well and can achieve segmentation with less resources compared to other models. Less resource consumption means it can reduce the amount of hardware computation and also the time required for training, so our method can run on less efficient hardware devices without the limitation of hardware environment.

### 3.3. Ablation Studies

Our proposed FPU-Net method, with the addition of the fine matching step after coarse prediction FPU, outperforms the original results in all evaluation metrics. To demonstrate the performance of the fine matching module, we used the fine matching module in different models, and the results in Table 6 clearly show that the fine matching module can indeed complement other types of models to produce more accurate results. This is because, after the fine matching module, a few extreme results can be corrected to make the overall evaluation value better.

## 4. Discussion

We propose a new femoral neck segmentation method, implemented in two steps. In the first step, we use a modified U-Net with feature propagation pathways, i.e., FPU-Net, to obtain coarse prediction results; in the second step, we employ a typical unsupervised learning K-means method with three specific parameters to fine-tune the coarse prediction results. Experiments on femoral neck segmentation were conducted to validate its feasibility, and typical segmentation networks, U-Net and PSPNet, were used as comparison algorithms. Due to the similarity of gray distribution in radio-graphic images, the U-Net method is not efficient enough to segment the region of the femoral neck, and extra or missed regions can be observed in the predicted results. Although PSPNet is able to overcome these problems, it still shows great prediction error.

Therefore, an FPU-Net for extracting more details of the original image with feature propagation, and which can suppress interference outside the target region, was created. The convergence rate of the FPU-Net is slightly slower by this newly added pathway, but both the U-Net and the proposed FPU-Net reach the same convergence. From the results of the coarse-predicted FPU-Net and U-Net, such as IOU values, the coarse-predicted FPU-Net method has made an obvious improvement.

In the second step, the K-means method was used to make a fine matching FPU-Net to further improve the results. Since K-means is unsupervised learning, it is convenient to classify the categories of the standard masks by setting the parameters of M, N and K. However, the more categories, the better the matching, but more computation time is required as well. Therefore, we should make a balance between accuracy and computing time to improve the network efficiency. In theory, a big dataset collecting various cases of patients will promote the performance of K-means. An augmentation of the dataset will help improve the efficiency of the network by rotation and scaling to simulate a variety of imaging cases, such as technical factors, as mentioned in previous sections, and the physical shapes of patients, the imaging orientations and so on. Meanwhile, the standard masks should be not less than 256 categories for the subsequent fine matching.

The method proposed in [28] for segmenting the thigh bone in CT images takes a traditional mathematical approach to segment the blocks. Because different tissues have different Hounsfield strength values in CT images, the surface is segmented into intact and fractured surfaces using different Hounsfield strengths. The study limitations occur because the method was tested on a Schatzker type VI fracture. In the case of even more complex fractures, the algorithm may require some improvements to identify all small fragments in order to fit.

In this study, we developed an innovative method that assists computer-aided diagnosis in the determination process of the exact location of the femoral neck junction in plain radiographs However, on X-ray images, the background and ROI region gray values are too similar, as shown in Figure 1; the border region of healthy bone tissue in X-ray film may show a gray distribution similar to that of fracture. Therefore, X-ray images cannot be solved by conventional mathematical modeling, so we proposed an accurate segmentation of the femoral neck junction region and labeled the ROIs to assist surgeons in computer-aided diagnosis. The limitations of this study can be discussed in two parts. First, senior orthopedic surgeons must mark the hip position on the radiographic image, which is very time consuming to accomplish. In addition, there are many radiographic technical factors, such as angulation, occlusion, and artifacts, that affect our ability to accurately acquire medical information and influence the accuracy of image segmentation.

## 5. Conclusions

Simulation results show that fine matching FPU-Net outperforms the classical U-Net and PSPNet method. The introduction of unsupervised learning of fine matching to rectify the results achieves an obvious improvement. A total of 2160 images were used to test our system and the values of IOU were improved over 25%. Our method requires 31%, 75% and 92% less resources than other methods, respectively, and can achieve segmentation with fewer resources than other models. In addition, the fine matching module can indeed complement other types of models to produce more accurate results. In conclusion, the proposed fine matching FPU-Net method, aided with unsupervised learning, can exclude the disturbs and accurately segment the femoral neck. Accurate segmentation of the femoral neck can assist the surgeon in diagnosis and reduce the burden and rate of misdiagnosis.

## Figures and Tables

**Table 1:** The detailed parameters of the encoder.

Module	Block	Layers	Size/Channel/Padding	Activation Function
Encoder	Stage1	AvgPooling	2*2	-
		Conv	3*3/64/same	ReLU
		Conv	3*3/64/same	ReLU
		BatchNormalization	-	-
		AvgPooling	2*2	-
	Stage2	Conv	3*3/128/same	ReLU
		Conv	3*3/128/same	ReLU
		BatchNormalization	-	-
		AvgPooling	2*2	-
	Stage3	Conv	3*3/256/same	ReLU
		Conv	3*3/256/same	ReLU
		BatchNormalization	-	-
		AvgPooling	2*2	-

**Table 2:** The detailed network parameters of FPP.

FPP	Stage1	AvgPooling	8*8	-
		Upsampling	8*8/64	-
		Conv	3*3/64/same	ReLU
	Stage2	AvgPooling	16*16	-
		Upsampling	16*16/128	-
		Conv	3*3/128/same	ReLU
	Stage3	AvgPooling	4*4	-
		Conv	3*3/256/same	ReLU
		AvgPooling	2*2	-

**Table 3:** The detailed parameters of the decoder.

Decoder	Stage1	Concatenate	-	-
		Conv	3*3/256/same	ReLU
		BatchNormalization	-	-
		Upsampling	2*2/128	-
	Stage2	Concatenate	-	-
		Conv	3*3/128/same	ReLU
		BatchNormalization	-	-
		Upsampling	2*2/64	-
	Stage3	Concatenate	-	-
		Conv	3*3/128/same	ReLU
		BatchNormalization	-	-
		Upsampling	2*2/64	-
		Conv	3*3/128/same	ReLU
		Upsampling	2*2/64	-
		Upsampling	2*2/32	-
		Conv	1*1/1/same	Sigmoid

**Table 4:** Calculation of IOU values.

Method	U-Net [18]	PSPNet [11]	Coarse Prediction FPU-Net	Fine Matching FPU-Net
IOU	0.5871	0.6400	0.6484	0.7586
	0.3600	0.5780	0.6273	0.8266
	0.5922	0.5497	0.6523	0.8258
Average	0.5137	0.5893	0.6426	0.8037

**Table 5:** Calculation of Evaluation Metrics.

Method	IOU	Dice	Hausdorff	Params
R2U-Net [25]	0.8388	0.9015	7.2216	10,958,321
Attention U-Net [26]	0.8307	0.8346	10.8084	29,858,965
R2AU-Net [27]	0.8726	0.9296	6.1503	96,035,437
Fine matching FPU-Net	0.8037	0.8602	11.1207	7,521,953

**Table 6:** Ablation Studies of Fine Matching.

Method	IOU	Dice	Hausdorff
R2U-Net	0.8388	0.9015	7.2216
R2U-Net [25] + Fine matching	0.8452	0.9058	6.8669
Attention U-Net [26]	0.8307	0.8346	10.8084
AU-Net [26] + Fine matching	0.8350	0.8996	10.5232
R2AU-Net [27]	0.8726	0.9296	6.1503
R2AU-Net [27] + Fine matching	0.9018	0.9437	6.1369
Coarse prediction FPU-Net	0.7512	0.8346	11.7684
Fine matching FPU-Net	0.8037	0.8602	11.1207

## Data Availability

The datasets used or analyzed during the current study are available from the corresponding author on reasonable request.
